# Subcarbonate of Iron in Tetanus
*Med. Chir. Trans. Vol. XV. Part I.


**Published:** 1829-10-01

**Authors:** 


SUBCARBONATE OF IRON IN TETANUS.*

That zealous and highly talented physician, Dr. Elliotson, has followed up his experiments on the powers of the subcarbonate of iron, and applied it to the treatment of tetanus. Of the original paper published in the first part of the thirteenth volume of the Medico-Chirurgical Transactions we have given an ample account to our readers, and it now remains to notice its present successor. The object of Dr. E. has been to prove that the subcarbonate might be given (in treacle) in much larger quantities than was previously imagined, and that even half-ounce doses were seldom productive of any inconvenience. The powers of the medicine in chorea were particularly pointed out, and reference was made to a case of traumatic tetanus, in which it appeared to effect a cure. The case in question is now detailed at length by Dr. Elliotson, so that the profession has an opportunity of judging for itself.

*Case.* Charles Frazier, middle-aged, received a compound dislocation of the great toe, December 10, 1825, and was carried to St. Thomas's Hospital. In the evening of the 24th, the lower jaw began to be stiff, and next morning the mouth could scarcely be opened sufficiently to allow the tongue to protrude. The bowels were ordered to be cleared by ℥ij. of the ol. terebinth. and then ℥ss. of the subcarbonate of iron to be taken every two hours. The dose of oil was repeated by the apothecary in the evening with the effect of procuring six evacuations, and the man not being able to swallow the subcarbonate, musk was substituted for it. Next morning the mouth was more closed, and the pulse 136. By diffusing each dose of the electuary, composed of the subcarbonate, to twice its weight of treacle, in a cup of beef tea, it was swallowed with ease, and regularly taken. The patient took also lbs. iij. of strong beef-tea daily. On the 26th, the trismus was rather increased, deglutition difficult, the abdominal muscles growing hard, the countenance anxious, the pulse 136. Pain had been felt in epigastrio, and there had been two stools with tenesmus. Half a pint of wine daily was added to the beef-tea. Next day the trismus was improved, and a little pus was evacuated from the foot; on the 28th, more matter was let out, and, the bowels being bound, an injection of colocynth administered after two common clysters had failed. On the 29th, the bowels were opened, the improvement decided, and the allowance of strong beef-tea increased to lbs. iv. On the 30th, the dorsum of the foot was very much inflamed, and on the 31st, he opened his mouth so wide that mutton chops were allowed him at his own

---

* Med. Chir. Trans. Vol. XV. Part I.

request. The recovery from the tetanus was so complete, that on the 7th the patient ceased to be under the care of Dr. Elliotson, and subsequently left the hospital completely cured. As the tetanic symptoms subsided, it is well to remark that the local injury produced more inconvenience, so that on the 2d and 3d, the foot was so painful as to deprive the man of rest.

The result of this case, in which the subcarbonate was given on principle, and seemed to produce such good effect, was highly gratifying to Dr. E. and determined him to apply it to the next example of tetanus it should fall to his lot to treat. Such an opportunity did not occur till after an interval of nearly three years, but on the 6th of November, 1828, Bryan Macguire, a labourer, aged 44, was admitted into George's Ward, labouring under trismus and opisthotonos. The mouth could only be half-opened; the muscles of the abdomen and back rigid; the body much arched, and during the spasms the pain at the epigastrium dreadful; the expression that of extreme agony; the pulse 76, regular, soft, and full. The thumb was slightly swollen and tender, and the nail was evidently separating, but without any pus below it.

Exactly a fortnight previous to admission he had jammed the thumb between two pieces of logwood, and torn the skin at the root of the nail, under which a little matter subsequently formed and escaped at one side. The trismus had begun six days before admission, the opisthotonos only one.

The bowels not having been opened for four days, were cleared by ℥ij. of ol. terebinth. and castor oil, after which the electuary of subcarbonate of iron diffused in strong beef-tea was exhibited every two hours, whilst lbs. iij. of beef-tea were also given daily. Seven stools were procured by the purge, but on the 7th, the symptoms were rather increased, and the dose of the iron was augmented to ʒiij. and a common injection of gruel, salt, and oil prescribed. Stools were again obtained, but no amelioration of the tetanic spasms, in consequence of which the dose was again raised on the 8th to ℥ss. of the subcarbonate, with a common injection. Next day the patient was still worse; a pint of milk and of porter were added to his diet, and the injection ordered to be administered twice a day. From the 9th to the 13th the patient continued in statu quo. Each injection coming away exactly in the same state in which it was administered, and being followed by several large dark red balls without pain, the dose of the medicine was diminished when the foregoing ceased to be the case. To ensure the facility of the discharge of these accumulations the injection was also ordered to be repeated three times a day. On the 13th the patient was somewhat better, and Dr. Elliotson discovered that above double the prescribed dose of the subcarbonate had been given. However, as it produced no inconvenience and the progress of the disease was evidently arrested, no alteration whatever was made. On the 14th, the improvement was decided, and from this time the medicine was not taken in the night. On the 17th, the opisthotonos was very slight, and the patient having suffered only four paroxysms in the previous forty-eight hours, the medicine was given only every four. On the 25th, he was so well that the medicine was discontinued in toto.

"I am ignorant," says Dr. E. "whether, if even the cure was effected by the remedy, a smaller quantity would not have been sufficient. I believe that in most cases of disease, doses of ʒi. or ʒij. two or three times a day, answer every purpose. But knowing as I did, that indefinite quantities are generally borne without inconvenience, and that large doses sometimes succeed after small ones have failed, and having a terrible disease to treat, which renders the system callous to many agents, and requires the full force of remedies to subdue it, I did not feel justified in trying whether less would accomplish the end. Among the large number of cases of tetanus upon record, I have read one only in which iron was exhibited, and then, together with many other means, the sulphate was given in doses of five grains every two hours, to a negress twelve years of age, for four days. She recovered.*

"Should iron prove a remedy in tetanus, I am aware that some cases will be too rapid for its action to be exerted, and that in others the degree of trismus will prevent its sufficient exhibition.

"But I dare not assert that these two cases were cured by the medicine, and I never before published upon the powers of a remedy, till long and careful experience had satisfied me that my results would be confirmed by all those who employed the remedy in the *same manner*, and under the *same circumstances* of the *same diseases* in which I had employed it. Yet when the facts formerly mentioned, of my having originally employed iron in tetanus from analogy; of the usual fatality of the disease, and the inefficacy of ordinary measures; of the gradual decline of the symptoms, in both my cases, soon after the commencement of the treatment; and of the magnitude of the quantities taken, are considered; together with the fact that these two successful cases are the only ones treated by me either in this way or in the Hospital at all, and the probability of a length of time elapsing before I meet with the disease again, I trust I shall be pardoned for communicating them to the Society."

We think that Dr. Elliotson displayed a very commendable caution in urging the claims of the subcarbonate in the wary and philosophical way he has done. So also the event went to prove; for in February of the present year, a patient was admitted into the hospital under the Doctor's care, in whom the remedy unfortunately failed. This case, with honourable and manly candour, a candour which deserves the more praise because of its scarcity in the present remarkable æra of falsehood, was instantly communicated to the Society, in the form of a postscript to the preceding paper.

The patient was 15 years of age, and admitted on the 17th, with the most dreadful tetanus and opisthotonos. A chilblain had existed over the right tendo achillis for about a fortnight, and had broken on the 8th; the trismus had begun on the 14th, and the opisthotonos on the 16th. The treatment pursued in the former cases was also had recourse to here, but at 8, p. m. of the following day a horrible spasm took place, and the patient died. On dissection, the brain and spinal marrow were found to be perfectly healthy, the lungs were gorged with blood, and several white spots were seen in the lining membrane of the aorta.

Dr. Elliotson does not consider that the case affords an argument against the use of the subcarbonate in tetanus, "any more than the former cases prove the efficacy of the medicine." The remedy in question exerts no influence in less than two or three days, and in this instance its exhibition was not commenced before the afternoon of the fourth, whilst the patient died on the fifth. We agree with Dr. E. in thinking that the question is yet sub judice, and earnestly implore our professional brethren to give the iron a trial in the cases of this melancholy disease it may fall to their lot to treat. For our own parts, we confess that our hopes of the ultimate value of the medicine are far from high, and we much fear that "acute tetanus" will continue to be, as it long has been, beyond the pale of medical art. Of the two successful cases related by the author, the first was clearly a mild case, such an one as frequently does well under various kinds of treatment. The second is a more satisfactory case, but how many circumstances step in to vitiate our conclusions in the practice of physic! We are all aware that numerous specifics

---

* "Memoirs of the Medical Society of London. Vol. VI. 1805. p. 77 et seq. The case was written in 1796, and the author does not lay any stress upon the iron. Oxyde of zinc, bark, opium, brandy, oleum succinatum, the hot bath, a clyster of tartarized antimony, and incision of a cicatrized burn followed by the application of caustic, were all simultaneously employed. But three exacerbations of spasm occurred after the application of the caustic."

have at different times been proposed with confidence for the cure of this disease, but where are they now? Buried with their authors in the tomb of the Capulets! By these remarks we would not wish to foreclose the question in the present instance, nor to shut the door against experiments with remedies. On the contrary, we again intreat practitioners to test the powers of the subcarbonate of iron in the manner recommended by Dr. Elliotson.

